# Early-Age Cracking of Fly Ash and GGBFS Concrete Due to Shrinkage, Creep, and Thermal Effects: A Review

**DOI:** 10.3390/ma17102288

**Published:** 2024-05-12

**Authors:** Yingda Zhang, Xinyue Liu, Ziyi Xu, Weiguang Yuan, Yong Xu, Zuobang Yao, Zihao Liu, Ruizhe Si

**Affiliations:** 1 School of Architecture and Civil Engineering, Xihua University, Chengdu 610039, China; yingda.zhang@mail.xhu.edu.cn (Y.Z.); liuxinyue1@stu.xhu.edu.cn (X.L.); 0120180009@mail.xhu.edu.cn (Y.X.); 2 Institute of Civil Engineering Materials, School of Civil Engineering, Southwest Jiaotong University, Chengdu 610031, China; xzy2020@my.swjtu.edu.cn; 3 School of Environment and Civil Engineering, Chengdu University of Technology, Chengdu 610059, China; yuanweiguang19@cdut.edu.cn; 4 Centre for Infrastructure Engineering and Safety, School of Civil and Environmental Engineering, The University of New South Wales, Sydney, NSW 2052, Australia; joe.zb.yao@outlook.com; 5 Department of Architecture, Faculty of Environmental Engineering, The University of Kitakyushu, 1-1 Hibikino Wakamatsu, Kitakyushu 8080135, Fukuoka, Japan; z-liu@kitakyu-u.ac.jp

**Keywords:** restrained shrinkage, early-age cracking, SCMs, degree of restraint, thermal cracking, model prediction

## Abstract

Supplementary cementitious materials (SCMs) are eco-friendly cementitious materials that can partially replace ordinary Portland cement (OPC). The occurrence of early-age cracking in OPC-SCM blended cement is a significant factor impacting the mechanical properties and durability of the concrete. This article presents a comprehensive review of the existing research on cracking in OPC-SCM concrete mix at early ages. To assess the effects of SCMs on the early-age cracking of concrete, the properties of blended cement-based concrete, in terms of its viscoelastic behavior, evolution of mechanical performance, and factors that affect the risk of cracking in concrete at early ages, are reviewed. The use of SCMs in OPC-SCM concrete mix can be an effective method for mitigating early-age cracking while improving the properties and durability of concrete structures. Previous research showed that the shrinkage and creep of OPC-SCM concrete mix are lower than those of conventional concrete. Moreover, the lower cement content of OPC-SCM concrete mix resulted in a better resistance to thermal cracking. Proper selection, proportioning, and implementation of SCMs in concrete can help to optimize the performance and reduce the environmental impact of OPC-SCM concrete mix.

## 1. Introduction

In the field of civil engineering, the shrinkage, creep, and temperature cracking of concrete are crucial issues that have a profound impact on the structural stability and durability of buildings. The causes and effects of these problems require in-depth understanding and research to effectively control and prevent them during the design and construction process.

Concrete shrinkage refers to the decrease in volume during the drying or hardening process. When concrete dries, the internal moisture gradually evaporates, resulting in a decrease in the concrete volume. This shrinkage is particularly evident during the early stages of concrete hardening and may result in surface cracks.

Creep refers to the deformation of concrete under long-term loading. Unlike elastic deformation, creep deformation is slow and increases with time. Creep can reduce the load-bearing capabilities of structures and may lead to a decrease in structural safety and stability.

Temperature cracks are caused by temperature changes in concrete. Due to poor thermal conductivity, when the temperature changes, there is an uneven temperature distribution inside and outside the concrete, leading to thermal expansion and contraction and ultimately the formation of temperature cracks. These cracks may affect the durability and waterproofness of the structure.

With the ongoing progress in science and technology, researchers have been searching for more environmentally friendly and efficient building materials. One increasingly popular option is the application of fly ash (FA) and slag to partially replace cement in concrete [1]. This new type of concrete not only has excellent engineering properties but also significantly reduces carbon emissions, contributing to the green development of the construction industry.

Fly ash and slag are both industrial waste products, but they can play unique roles in concrete. Fly ash, a fine ash collected from the smoke of coal combustion, has high volcanic activity and can partially replace cement while enhancing the density of concrete. Slag, on the other hand, is a waste product produced during manufacturing that can be used as a binder in concrete after proper treatment. This not only reduces the production cost of concrete but also achieves the resource utilization of waste products.

The application of fly ash and slag to replace some of the cement in concrete has significant advantages. First, this type of concrete has higher strength and durability [2,3]. Due to the characteristics of fly ash and slag, the internal structure of concrete is denser than that of the other materials, decreasing susceptibility to external environmental factors and improving durability. Second, the production process of this type of concrete has lower carbon emissions. Compared to traditional cement production processes involving significant carbon emissions, using fly ash and slag significantly reduces carbon emissions during concrete production, which aligns with the green building development trend. Finally, this type of concrete has better economic benefits. The production cost of concrete can be reduced, and the utilization rate of waste products can be improved by using industrial waste products to partially replace cement, achieving a win–win situation for both economic and environmental benefits.

However, using fly ash and slag to replace cement in concrete also comes with some challenges. For example, ensuring the quality and stability of concrete is a crucial concern. Additionally, the rational utilization of waste products to avoid secondary pollution to the environment is also a consideration. Therefore, when using this new type of concrete, it is necessary to combine specific project needs with actual circumstances and implement reasonable blending and quality control measures.

Fly ash and GGBFS can lead to different cracking risks compared to conventional concrete. For instance, Gao et al. [4] conducted a self-designed elliptical ring test and the results showed that introduction of fly ash can effectively improve the crack resistance of concrete. Similar results were also found by Wang et al. [5], who demonstrated that the effect of the pozzolanic reaction of fly ash was of benefit to the thermal cracking resistance of mass concrete. For GGBFS concrete, Liu and Ma [6] illustrated that the cracking risks of GGBFS concrete were lower than those of OPC concrete because GGBFS can fill the space among the cement particles, leading to a reduced surface area of the interfacial transition zone (ITZ).

Moreover, researchers have explored the impact of supplementary cementitious materials (SCMs) on various concrete properties, including mechanical strength, shrinkage, and creep characteristics. However, there is limited documentation on the evaluation of cracks in concrete incorporating SCMs at early ages. The occurrence of cracking in an OPC-SCM concrete mix at early ages is a multifaceted phenomenon influenced by factors like the mechanical properties, thermal gradient, restraint, shrinkage, and creep characteristics of the concrete [7]. The existing review articles mainly focus on discussing the behavior of early-age cracking of OPC concrete [8,9,10]. As such, a comprehensive review of cracking of SCMs blended with cementitious materials at early ages is lacking. This paper presents relevant research studies on the cracking of cementitious materials at early ages and identifies the gaps in the existing literature, as well as the proposed methodology for further research.

## 2. Supplementary Cementitious Materials (SCMs)

### 2.1. Fly Ash

Fly ash (FA) is an industrial waste material with volcanic ash activity that can be used as an admixture in concrete to improve its performance. When fly ash is added to concrete, its performance undergoes a series of changes. First, the flexural strength and compressive strength of concrete mixtures can be enhanced by fly ash. This is due to the small particles of fly ash filling the gaps between cement particles, which densifies the concrete and thereby improves its mechanical properties. Second, fly ash can reduce the drying shrinkage of concrete, improving its durability and crack resistance. This is because fly ash can absorb excess moisture in concrete and form a dense film that effectively prevents moisture evaporation and the erosion of harmful substances, thereby enhancing the durability of the concrete [11,12,13]. As such, fly ash can increase the autogenous shrinkage [14], but the total shrinkage of fly ash concrete is comparable to that of OPC concrete due to the compensation of low drying shrinkage [15].

Furthermore, fly ash enhances concrete durability by bolstering resistance against sulfate attack and inhibiting chloride ion penetration. This is because certain chemical components in fly ash can react with sulfates and chloride ions, reducing their erosive effect on concrete and improving the density of the microstructure [16].

Fly ash can notably diminish the early-age heat of hydration, attributed to a dilution effect, resulting in a reduced risk of thermal cracking [17,18]. Dockter [19] suggested that FA concrete could mitigate the volume changes due to the alkali–silica reaction (ASR). The ASR mitigation of fly ash in concrete is influenced by alkali binding, diffusion control, and reduction in available Ca(OH)_2_ [20]. Ling et al. [21] observed a slower strength development in FA concrete compared to normal concrete. Additionally, Naik et al. [22] reported that the permeability and porosity of concrete could be reduced by incorporation of FA in the mixtures.

Fly ash alters the early-age behavior of concrete at the microscopic level. Fládr et al. [23] reported that the increase in the level of fly ash replacement led to an increase in micro-porosity, and fly ash reduced the presence of low-density C-S-H but increased that of high-density C-S-H. When fly ash was incorporated, the concrete pore structure was influenced due to the pore-filler effect and densification due to the pozzolanic reaction. The cumulative pore volume in fly ash concrete notably rises within the medium capillary pore zone [24].

As such, when using fly ash in concrete, issues such as activity requirements, particle size distribution, dosage control, curing conditions, and storage and transportation need to be considered. Selecting fly ash that meets the requirements and comprehensively considering various performance indicators of the concrete can improve the performance and stability of the concrete [25].

### 2.2. Slag

Slag is a common industrial waste that can be used as an admixture in concrete to improve its performance. When slag is added to concrete, its performance undergoes a series of changes [26]. First, slag contributes substantially to the enhancement of both flexural and compressive strength in concrete. This is due to the small particles of slag filling the gaps between cement particles, which densifies the concrete and thereby improves its mechanical properties. In addition, certain chemical components in slag can react with certain components in cement to form a stronger crystal structure. Second, slag can enhance the toughness and crack resistance of concrete [27,28]. This is because slag can absorb excess moisture in the concrete and form a dense film that effectively prevents moisture evaporation and the erosion of harmful substances, thereby enhancing the durability of the concrete. In addition, the small particles of slag can also form small voids in the concrete, which can relieve stress concentration and thereby improve the toughness of the concrete [29].

Regarding the microscopic behavior of GGBFS-based concrete, Zhang et al. [30] illustrated that the addition of GGBFS can decrease the content of portlandite and improve the compactness of the ITZ, as well as refine the pore structure of concrete. Yalçınkaya and Çopuroğlu [31] reported that GGBFS can lead to elephant skin formation of UHPC because most of the air bubbles of UHPC cannot escape from the fresh cement matrix.

Furthermore, slag can also improve concrete durability by bolstering resistance against sulfate attack and inhibiting chloride ion penetration. This is because certain chemical components in slag can react with sulfates and chloride ions, reducing their erosive effect on the concrete. According to the work of Li et al. [32], the hydration reaction of ground granulated blast furnace slag (GGBFS) occurs at a slower rate compared to that of cement, resulting in a prolonged setting time.

## 3. Time-Dependent Behaviors

### 3.1. Autogenous Shrinkage

Concrete autogenous shrinkage refers to the decrease in volume during the hardening process due to chemical reactions and capillary action. This type of shrinkage typically begins immediately after concrete placement and may continue over a period. The autogenous shrinkage of ordinary concrete is affected by different factors, such as the aggregate content and type, water-to-cement (w/c) ratio, and type of cement. This process is influenced by many factors, including environmental humidity, temperature, and concrete curing conditions [33]. Autogenous shrinkage is regarded as an inherent aspect of shrinkage as the reduction in volume takes place without the concrete losing moisture to the surrounding environment [34].

However, when certain additives are added to concrete, such as FA and GGBFS, the nature and degree of autogenous shrinkage may change. The effect of fly ash on autogenous shrinkage is still being debated. First, it was reported that incorporation of FA can mitigate autogenous shrinkage in high-performance concrete (HPC) [35]. Yan and Chen [36] stated that autogenous shrinkage of fly ash concrete quasi-linearly decreased with the increase in the addition of fly ash. The results showed that the greater the FA substitution rate, the less autogenous shrinkage occurred. This is mainly because fly ash can fill the capillaries in concrete, reducing moisture evaporation and thus reducing autogenous shrinkage. Some studies reported that fly ash can increase autogenous shrinkage. For example, according to the study of Termkhajornkit et al. [14], FA exerted a notable impact on autogenous shrinkage during the early ages but had only a minimal effect at later ages.

**Figure 1:**
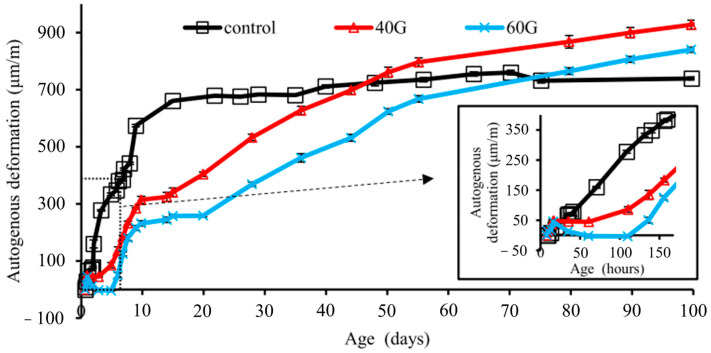
Influence of GGBFS on autogenous shrinkage [37].

On the contrary, GGBFS yielded contrasting outcomes regarding autogenous shrinkage compared to FA. As illustrated in Figure 1, GGBFS concrete exhibited a late reaction and contributed to higher autogenous shrinkage than the reference concrete [37]. The HPC containing GGBFS tended to enhance autogenous shrinkage [38]. Lim et al. [39] observed accelerated compressive strength development and heightened autogenous shrinkage in concrete mixtures containing GGBFS. Wei et al. [40] asserted that GGBFS has the potential to decrease early-age shrinkage due to the delayed pozzolanic reactions compared with the cement hydration; however, in the long-term, autogenous shrinkage surpassed that of the plain concrete due to sustained reactions involving GGBFS.

### 3.2. Drying Shrinkage

Concrete drying shrinkage refers to the decrease in volume of concrete after hardening caused by moisture evaporation. This type of shrinkage typically begins within a period after concrete placement and may continue for a period of months or even years [41]. The drying shrinkage of ordinary concrete is affected by different factors, including the water-to-cement ratio, type of cement, and aggregate type and content. This process is influenced by many factors, including environmental humidity, temperature, and concrete curing conditions.

Extensive research has been conducted on the drying shrinkage of concrete mixes containing FA and GGBFS. Drying shrinkage of the concrete can be effectively reduced with the increased FA replacement ratio in the mixture [41]. It was reported that incorporation of 40% of FA in concrete can reduce drying shrinkage by up to 20% after 180 days of drying [41]. Comparable findings were presented by Seo et al. [42], who emphasized the advantageous reduction in drying shrinkage for mitigating the risk of concrete cracking under restrained conditions. The drying shrinkage reduction effect of FA is mainly because FA can fill the capillaries in concrete, reducing moisture evaporation and thus reducing drying shrinkage.

In terms of GGBFS, Yuan et al. [43] concluded that GGBFS-containing concrete tends to have lower drying shrinkage than a pure cement mixture. The decline in drying shrinkage became more pronounced with an increase in the level of cement replacement with GGBFS. This phenomenon is attributed to the slag particles, which can effectively occupy the pores in the concrete, mitigating capillary strength and consequently decreasing drying shrinkage. In the study by Saluja et al. [44], the investigation focused on the drying shrinkage behavior of GGBFS-containing concrete exposed to varying temperature conditions. The findings revealed a direct correlation with drying shrinkage temperature, as temperature significantly influences water loss.

For exposed concrete surfaces without sealing, the speed of moisture dissipation relies significantly on external conditions like airflow, humidity, sunlight exposure, and surrounding temperature [45]. In the case of substantial concrete structures prone to early-age thermal and autogenous distortions, the impact of drying shrinkage strains is typically less pronounced in the initial days post-concrete placement. Ensuring appropriate curing methods and adhering to sound construction practices becomes crucial in curtailing the repercussions of confined drying shrinkage [46,47]. Moreover, incorporating substances like fly ash or slag can contribute to alleviating these consequences [48].

### 3.3. Creep

The phenomenon where the deformation of concrete progressively intensifies over time under a sustained, long-term load is known as concrete creep. This deformation initiates promptly after the casting of concrete and can endure for numerous months or potentially extend over several years. The creep behavior of concrete is primarily associated with time, typically exhibiting a rapid increase in the early stage followed by a gradual decline, reaching stability within 2 to 5 years [49,50]. In situations where concrete shrinkage encounters restriction, tensile creep emerges, resulting in tensile stress relaxation. This aspect requires consideration while the tensile stress needs to be calculated.

Extensive research has been conducted on the creep performance of concrete incorporating FA and GGBFS. In terms of the difference of the creep behavior of concrete with the added FA and GGBFS, broadly speaking, the inclusion of fly ash tends to diminish concrete creep, whereas the incorporation of slag may increase the creep. Zhao et al. [51] investigated the creep properties of the concrete with FA under varying curing temperatures. Their findings revealed that the microstructure of C-S-H gel and reaction degree of the raw materials played a critical role in influencing the creep behavior of FA concrete. This is primarily because FA can fill the capillaries in concrete, mitigating moisture evaporation and thereby decreasing creep.

**Figure 2:**
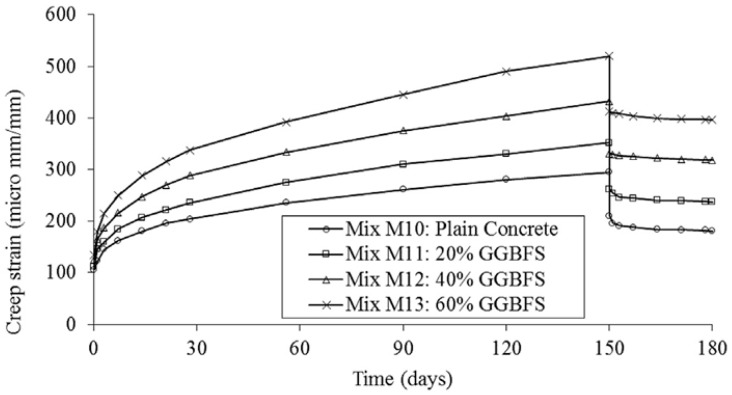
Effect of GGBFS on creep [54].

Wei et al. [52] reported that the evolution of tensile strain and stress of the concrete containing GGBFS was more rapid and substantial under higher curing temperatures. The influence of GGBFS was not pronounced within a 12-day testing period [52]. Khan et al. [53] found that the tensile creep strength of GGBFS-containing concrete exceeded that observed in cement-only samples at early ages. Similar findings were reported by Shariq et al. [54]. The presence of micro-spherical slag particles, which can fill the pores in concrete and reduce capillary action, might contribute to an escalation in concrete creep, as shown in Figure 2.

### 3.4. Thermal Effects

Temperature-induced cracks are the cracks that are presented both internally and on the concrete surface as a consequence of fluctuations in temperature. These cracks typically emerge during the hardening process of concrete and are primarily caused by factors such as internal and external temperature differences and cement hydration heat [55]. The shape and size of temperature cracks vary, potentially impacting the structural strength and durability of concrete [56].

**Figure 3:**
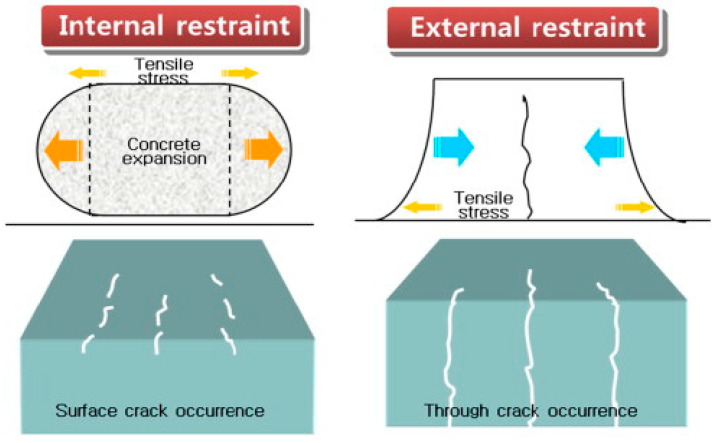
Schematic of the thermal crack generation in concrete [57].

Since the thermal conductivity of the concrete is low, the enhanced internal temperature of the concrete caused by the hydration of cement can result in the thermal gradients between the surface and interior of concrete [55]. The tensile stress can be introduced by the thermal gradients, potentially causing surface cracks, while the element’s interior experiences compressive stress. When the environment temperature is lower than that of the surface of the concrete, the interior encounters tensile stress due to the external constraints and thermal gradient. This scenario may result in the development of penetrating cracks. Figure 3 illustrates the mechanisms behind cracking induced by temperature variations in concrete.

The formation of cracks in concrete due to temperature is closely connected to the deformations induced by temperature variations in the concrete. The water-binder ratio, and content and type of cementitious material, affect the quantity of generated heat of the mixture. The thermal strain resulting from temperature changes is determined by the concrete’s coefficient of thermal expansion (CTE). Therefore, the CTE serves as a crucial parameter in concrete, offering a method for calculating thermal strain and facilitating precise determination of thermal stress. The CTE refers to the rate of change in the concrete length or volume per unit temperature change [58]. In the work of Kada et al. [59], the significant variations in the CTE of curing concrete within initial hours after the casting of the concrete were observed. Consequently, when assessing the behavior of thermal stress of concrete in its early stages, depending on an unchanged CTE value calculated for fully cured concrete is unreliable. Neglecting to consider changes in CTE during the early stages may lead to an undervaluation of temperature-induced stress evolution.

Regarding the changes in concrete temperature cracks after adding fly ash and slag, generally speaking, fly ash can reduce concrete temperature cracks, while slag may increase concrete temperature cracks. This is mainly because fly ash can lower the hydration heat of concrete, thereby reducing the generation of temperature cracks. Slag, due to its micro-spherical particles, can fill the pores in concrete, reducing capillary action and potentially increasing temperature cracks. Moreover, it has been reported that GGBFS particle hydration is highly related to the ettringite formation because of aluminate phases for ettringite formation, leading to the long setting time and low early-age mechanical properties and hydration heat flow [60].

### 3.5. Mechanical Properties

**Figure 4:**
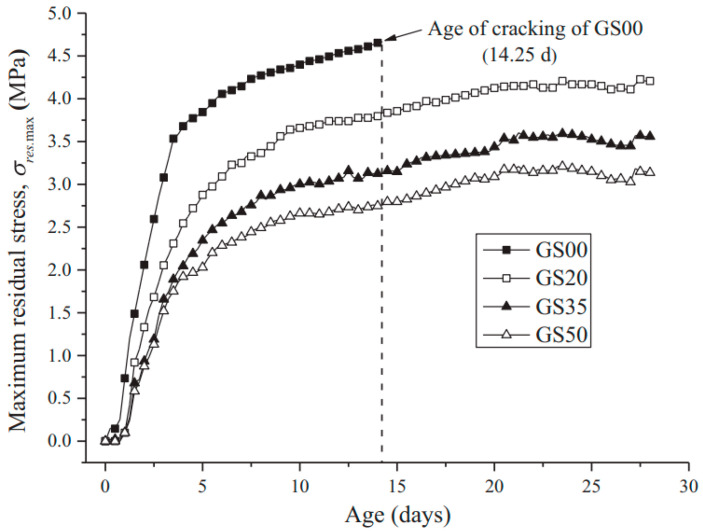
Residual stress development of OPC concrete and GGBFS concrete [65].

The emergence of tensile stress from restricted shrinkage is influenced by both deformation and evolution of mechanical properties, such as modulus of elasticity, characteristics of tensile relaxation, the extent of external constraint, and tensile strength. Moreover, in the case of mass concrete experiencing a substantial temperature rise from cement hydration, especially within 24 to 36 h post-placement, the mechanical properties of concrete, particularly the modulus of elasticity, exhibit rapid development [61]. Research findings from ref. [62,63] indicate that the increase in the elastic modulus surpasses that of tensile strength during this period, suggesting a notable escalation in tensile stress. Nevertheless, investigations into stress progression in concrete at early ages have highlighted the importance of concrete’s viscoelastic behavior, which can lead to substantial stress relaxation. Previous studies suggested that up to 60% of the stress generated from shrinkage can be alleviated by tensile relaxation [63,64]. As shown in Figure 4, it has been reported that the residual stress of concrete reduced with increasing GGBFS replacement [65]. The above discussion emphasizes the necessity of comprehensively examining the behavior of concrete while investigating the stresses induced by restrained shrinkage, including factors like tensile creep, free shrinkage, susceptibility to cracking, and temperature elevation. Specifically, the evolution of mechanical properties has to be performed with a temperature history akin to that of the core section of the concrete element.

As previously mentioned, comprehensively examining concrete behavior is necessary when investigating the evolution of tensile stresses induced by restrained shrinkage. This examination should consider temperature elevation, tensile creep, shrinkage, and sensitivity of cracking. In particular, the assessment of mechanical properties has to be performed under a temperature history akin to that experienced by the core section of the concrete.

## 4. Factors Influencing Cracking in Concrete

### 4.1. Degree of Restraint

In concrete restrained cracking, the degree of constraint refers to the degree to which external factors restrict the free deformation of the concrete. These constraints can be caused by factors such as internal shrinkage, external loads, and temperature changes.

This constraint is closely related to the cracking behavior of concrete [56,66,67]. When the constraint on concrete is low, the free deformation is large, and the concrete is less likely to crack. As the constraint increases, the free deformation of the concrete is restricted, increasing the susceptibility to cracking. Furthermore, the generated stress is a combination of distinct stress components arising from external and internal constraints. The stress from internal constraints originates from the uneven distribution of temperature inside the concrete elements. The stress from external restraints can be generated as the contraction or expansion of a structural element encountering impediments from foundations, surrounding structures, and the underlying subsoil. The magnitude of external restraint is mainly contingent upon the comparative modulus of elasticity and dimensions of both the restraining elements and concrete.

**Figure 5:**
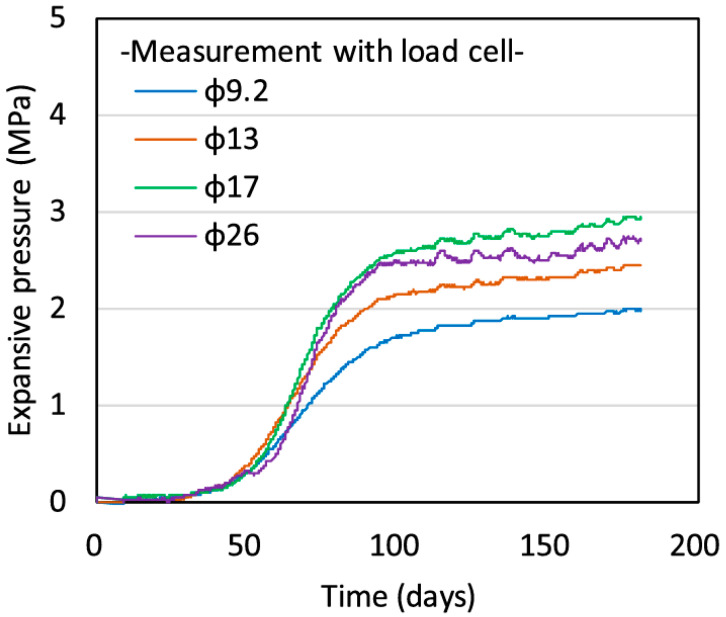
Influence of expansive behavior and degree of restraint [68].

Kawabata et al. [68] examined the impact of the restraint degree on the patterns of cracking and expansive pressure in concrete. Their observations indicated a significant correlation between expansion behavior and the restraint degree, as shown in Figure 5. Zych [69] investigated the external constraint of wall elements in reinforced concrete. In engineering practice, the degree of constraint can be adjusted by controlling factors, such as the mix ratio of concrete, curing conditions, and load conditions, to avoid or mitigate concrete cracking. The cracking of concrete is related not only to the degree of restraint, but also to other factors, such as the material properties of the concrete and the environmental conditions. Therefore, in practical engineering, various factors need to be comprehensively considered based on specific situations to prevent or mitigate concrete cracking.

### 4.2. Effect of Concrete Constituents

The mechanical properties of concrete can be negatively affected by the added SCMs (such as FA and GGBFS) at early ages. Consequently, the occurrence of cracking in concrete at early ages is notably influenced by the type of cementitious materials [70]. Altoubat et al. [71] investigated the influence of fly ash on early-age cracking by performing a restrained shrinkage test. The findings indicated that the relaxation behavior and cracking resistance of the mixture can be enhanced by introduction of FA compared to a cement-only mixture. Furthermore, the curing conditions, including air curing, 3-day water curing, and 7-day water curing, also play a role in determining the potential for cracking. The application of water curing can notably reduce cracking at early ages compared to air curing alone. Regarding concrete with GGBFS, Shen et al. [72] asserted that the addition of 50% slag can mitigate the restrained strain, resulting in improved cracking resistance compared to that observed in the cement-only mixture.

The selection of the binding materials determined the temperature evolution of the mass concrete. The previous literature [73,74,75,76,77] has indicated that the use of ASTM Type II or Type IV cement, as well as partial replacement of OPC with SCMs, tends to markedly diminish the temperature elevation in concrete compared with the concrete made by Type I cement. Furthermore, elevating the w/b ratio results in a decrease in the hydration heat and decelerates the release rate of the heat during the early-age hydration.

**Figure 6:**
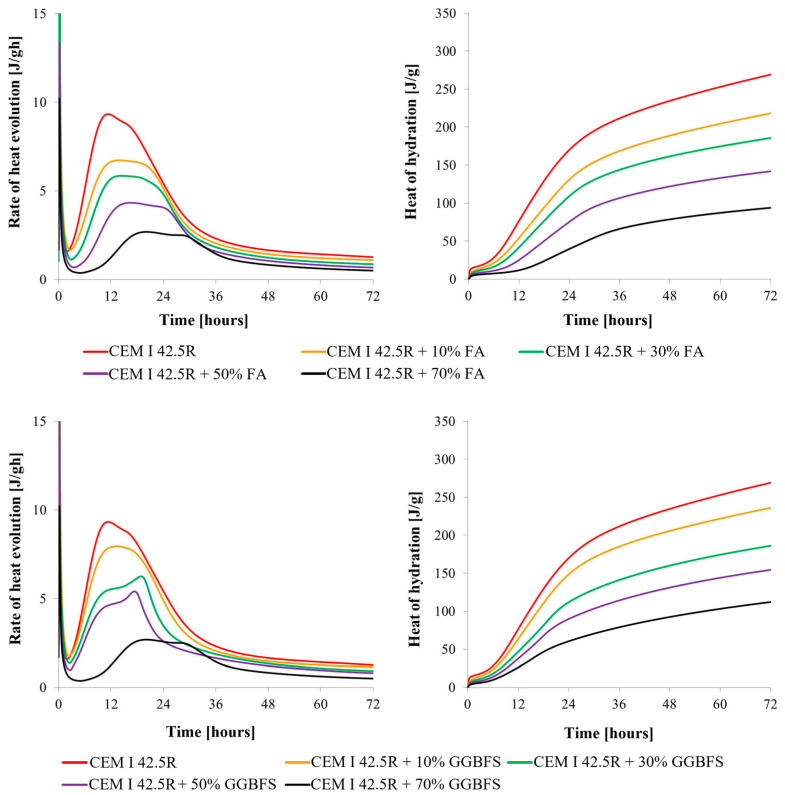
Influence of siliceous FA and GGBFS on the hydration heat and heat release rate under isothermal conditions (20 °C, w/c = 0.5 [76].

Batog and Giergiczny [76] investigated the impact of incorporating FA and GGBFS into cement on the hydration heat and hardening temperature of concrete. The outcomes of their investigation are depicted in Figure 6. As the proportions of FA or GGBFS in the cement mixtures increased, there was a notable reduction in both the quantity and rate of heat generated. This effect was particularly obvious in mixtures containing FA.

Despite the potential for SCMs to mitigate the rise of temperature in concrete structures, the occurrence of cracking remains a possibility. As noted by Springenschmid et al. [61], this is attributed to the slow development of the elastic modulus in the initial hardening stage, specifically within the first 12 h. Consequently, there is insufficient formation of compressive stresses within this timeframe to counteract the tensile stresses arising at the subsequent cooling stage. Furthermore, the decreased hydration rate leads to a lower tensile strength, which hinders the impact of thermal stresses.

As previously mentioned, further investigation is needed to explore the impact of SCMs on both total shrinkage and the evolution of mechanical properties, including strength, modulus of elasticity, heat evolution, and creep coefficient in mass concrete.

## 5. Analytical Models That Predict Factors Affecting Early-Age Concrete Cracking

### 5.1. Analytical Models for Predicting Shrinkage

The shrinkage behavior is a highly intricate physical and chemical process, influenced by numerous factors. Material-related aspects, including the quantity and type of cement, content of aggregate, and w/c ratio, as well as the type and quantity of SCMs and admixtures, exert a substantial impact on shrinkage. Construction and environmental considerations, such as the relative temperature surrounding the concrete structure, play a pivotal role in affecting shrinkage. Structurally, factors like nominal section size and volume-to-surface ratio, among others, bear a significant influence on drying shrinkage. Therefore, to quantitatively estimate concrete shrinkage, it is also necessary to comprehensively consider the above three aspects.

The ACI209-92 model proposed by The American Concrete Institute (ACI) in 1992 was subsequently reconfirmed and adopted in 2008 [78]. This model considers numerous factors, including the content of sand, type and content of cement, porosity, slump, duration and temperature of curing, curing humidity, temperature of concrete, content of water, and shape and size. The European Concrete Commission and the International Prestressed Concrete Association proposed the fib Model Code 2010 model [79]. This model accounts for the age of the concrete element at the beginning of drying, duration of drying, concrete age, 28-day compressive strength, type of cement, notional size, relative humidity, etc. Bazant et al. derived statistics and conducted analysis based on the B3 model and proposed a more applicable B4 model in 2015 [80]. In the B4 model, the autogenous and drying shrinkage models are split. The B4 model considers the aggregate type, effective thickness, cement type, elastic modulus, relative humidity, shape, age of concrete at the beginning of drying, etc. Note that this model also applies to concrete-containing admixtures. In 1993, the GZ 1993 model was introduced by Gardner and Zhao, formulated through an analysis of numerous results from long-term shrinkage tests; subsequently, Gardner and Lockman proposed the GL 2000 model by improving the GZ 1993 model [81]. The GL 2000 model requires the 28-day compressive strength, element size, and relative humidity as inputs. This model can be used regardless of the type of admixture, temperature, or curing regime.

In 2018, a shrinkage prediction model was introduced by the Australian Concrete Association AS3600 [82]. This model requires compressive strength, time, location factor, notional size, etc., as inputs. The shrinkage prediction model introduced by the European Concrete Association in the Eurocode 2 model [83] considers the type of cement, relative humidity, notional size, compressive strength, etc. Table 1 summarizes the parameters needed for different prediction models.

Goel et al. [84] compared shrinkage test results and prediction models for various grades of concrete. They found that the shrinkage prediction by the GL 2000 model was the closest to the corresponding experimental results. Al-Manaseer and Prado [85] summarized and analyzed the RILEM and NU-ITI concrete databases and concluded that the ACI209R model was the closest to the test results, followed by the B3 and fib 2010 models. Shariq et al. [54] compared various models and shrinkage test results for slag concrete and found that the ACI209-R92 model had the lowest prediction results, while the GL2000 model had the highest prediction results. Kataoka et al. [86] also conducted a shrinkage test and reported that the ACI209R and GL 2000 models provided acceptable predictions. However, these prediction models are calibrated for OPC-based concrete instead of FA- and GGBFS-based concrete. As a result, additional shrinkage tests should be performed on the basis of the SCM-based concrete.

### 5.2. Analytical Models for Predicting Creep

The choice of a creep prediction model for concrete is related to the accuracy of the creep prediction values. Researchers worldwide have proposed different creep prediction models, but these models are all empirical formulas based on many experiments.

The ACI 209R model [78] can estimate the ultimate creep value by employing a hyperbolic function and expressing the computed value as a function of time. This model can calculate the creep of Type I and Type III cement under the relative humidity range from 40% to 100%, considering both steam and standard curing conditions. The concrete age at loading, sample size, surrounding temperature, relative humidity, conditions of curing, and the portion of the fine aggregate are the main factors determining the shape and limit of the curve.

The fib Model Code 2010 model [79] emerged from a multitude of experimental tests. It can be applied to concrete under ambient temperature (5–30 °C) and relative humidity levels from 40% to 100%, with a strength within the range of 12 to 80 MPa. The applied maximum strength has to be lower than 40% of the compressive strength. This model is divided into two classifications, namely basic creep and drying creep. The compressive strength, specimen size, ambient relative humidity over time, etc., are considered. The model also innovatively proposes that the temperature effect of concrete is equivalent to the adjusted age of concrete, and the adjusted age is used to predict the creep of the concrete.

In 1995, Bazant et al. [87] established the B3 model and divided creep into two classifications: drying creep and basic creep. They deduced the calculation expressions of drying creep and basic creep through consolidation theory and diffusion theory. Thus, this model is a semi-empirical and semi-theoretical formula according to theoretical and experimental data. The B3 model considers the influence of the w/c ratio, cement type, cement content, coarse and fine aggregate contents, concrete drying period, ambient relative humidity, concrete density, age of concrete at loading, etc. The B3 model also calculates the concrete creep strain through the compliance function, which is divided into the elastic compliance function, basic creep compliance, and drying creep compliance. Nevertheless, this model is usually not suitable for early-age creep. Østergaard et al. [88] introduced the parameter q5 to adjust viscoelastic parameter 2 of the B3 model to obtain the modified early-age creep model. D’Ambrosia [89] also affirmed the suitability of the modified B3 model for early-age concrete through performing the early-age creep tests.

On the other hand, the B4 model can be applied to estimate the creep of more types of concrete, such as concrete mixes with FA, silica fume, superplasticizer, air-entraining agent, and other admixtures, while the effect of admixtures on creep has not been considered by other models [80]. Moreover, a simplified model was also proposed, and concrete creep can be predicted accurately when only the 28-day compressive strength of the concrete is known.

The GL2000 model [81] takes into account various factors, including relative humidity, volume/surface ratio, duration of loading, initial loading, and drying period of concrete. This model offers enhanced convenience in calculation as it does not differentiate between basic and drying creep, ensuring accurate calculations.

The AS3600-2018 model [82] takes into consideration the total creep without separate calculations for basic and drying creep. The strength factor, creep factor, age of loading thickness, location, temporal development of creep are considered in this model.

For the Eurocode 2 model [83], the relative humidity, the time of loading, nonlinear creep factor, compressive strength, and temporal development of creep are considered. This model can be employed for predicting the creep of concrete under ambient temperature from −40 to 40 °C and relative humidity from 40% to 100%. Table 2 summarizes the parameters needed for the different prediction models.

Goel et al. [84] compared creep tests and prediction models for various grades of concrete. They found that the compressive creep prediction by the GL 2000 model was the closest to its experimental results. Al-Manaseer and Prado [85] summarized and analyzed the RILEM and NU-ITI concrete databases and concluded that the ACI209-R92 model was the closest to the test results, followed by the B3 and GL2000 models. Shariq et al. [54] compared various models and compressive creep test results for slag concrete and found that the ACI209-R92 model had the lowest prediction results, while the GL2000 model had the highest prediction results.

The above models do not specify whether they can be used to predict compressive or tensile creep. However, these models were deduced based on compressive creep test results. According to the previous discussion, the mechanisms and characteristics of tensile and compressive creep are different under a certain stress level. As such, if the above models are used to predict tensile creep directly, considerable deviations and variations between the prediction and test results will be observed.

Dabarera et al. [90] measured the tensile creep of HPC employing TSTM and dog-bone samples and compared the test results with the basic creep model of the fib Model Code 2010. The findings showed that the fib Model Code 2010 model significantly underestimates the tensile basic creep of HPC at early ages. Thus, they introduced a modified basic tensile creep model derived from the basic compressive creep model of the fib 2010 model. The validity of the proposed model was affirmed through validation against experimental results conducted by Ji et al. [91] and Atrushi [92]. Zhang et al. [93] proposed a tensile creep model for FA and GGBFS concrete based on specific test conditions (23 °C and 50%). They successfully verified the applicability of the proposed model for estimating tensile stress during the restrained shrinkage ring test.

There are still many concerns to be addressed in the study of tensile creep models: (1) The establishment of a compressive creep model is based on experimental statistical data, the statistical data of tensile creep are insufficient, and the uncertainty of the model is difficult to measure quantitatively. (2) The linear creep coefficient model constitutes a binary function characterized by two parameters: the initial loading age and the load holding time. However, the comprehensive consideration of the influence of the initial loading age on the creep model remains incomplete. (3) The size effect of tensile creep and the influence of temperature and humidity parameters still lack systematic research.

### 5.3. Analytical Models for Predicting Evolution of Tensile Stress during the Restrained Ring Test

Normally, the cracking of concrete due to the constrained shrinkage at early ages can be tested by using three test methods: the restrained ring test (RRT) [94,95], the restrained shrinkage test in one dimension [96,97], and the restrained shrinkage test in two dimensions [98,99]. Among these approaches, the RRT is the most frequently utilized experimental technique to assess the susceptibility of concrete to cracking under constrained shrinkage. This preference arises from its practical implementation compared to the other two test methods [100,101,102].

Hossain and Weiss [100] introduced a method to assess both the evolution of stress relaxation and residual stress through the RRT. Both residual stress and theoretical elastic stress can be quantitatively estimated using their approach. To study the influence of tensile creep on cracking performance of concrete at early ages, Khan et al. [66] used both the tensile creep test and RRT. Their findings revealed that the coefficients of tensile creep obtained from tensile creep tests can effectively be applied to assess the tensile creep-induced stress relaxation during the RRT.

The method of computing tensile stress evolution in concrete, based on testing the strain of the steel of the steel ring, is not suitable for predicting such development and can only be used to analyze the results of ring tests [100]. Moreover, the theoretical elastic stress model proposed by Hossain et al. [100] enables the estimation of concrete tensile stress based on the restraint degree, modulus of elasticity, and free shrinkage, but does not consider the effect of tensile creep. To address this limitation, Zhang et al. [67] introduced a model to analyze the RRT that captures the effects of tensile creep and restrained shrinkage using the method of the age-adjusted effective modulus, as shown in Equations (1) and (2). According to experimental data capturing the time-dependent evolution of the tensile creep, total free shrinkage, and modulus of elasticity, the analytical model effectively forecasts the tensile stress in restrained concrete rings. The model’s accuracy and reliability were confirmed through successful validation using numerical finite element simulation.

(1) $$\sigma_{cs}=D_{R}\;\varepsilon_{sh}\;{\bar{E}}_{e}$$

where $\sigma_{cs}$ is the tensile stress experienced by the restrained concrete rings, $D_{R}$ is the restraint degree and can be estimated using Equation (2), and ${\bar{E}}_{e}$ is the age-adjusted effective modulus.

(2) $$D_{R}=\frac{\frac{R_{OC}^{2}+R_{OS}^{2}}{R_{OC}^{2}-R_{OS}^{2}}}{\frac{{\bar{E}}_{e}}{E_{s}}\cdot \frac{\left[\left(1+\nu_{s}\right)R_{IS}^{2}+\left(1-\nu_{s}\right)R_{OS}^{2}\right]}{\left(R_{OS}^{2}-R_{IS}^{2}\right)}+\frac{\left[\left(1+\nu_{c}\right)R_{OC}^{2}+\left(1-\nu_{c}\right)R_{OS}^{2}\right]}{\left(R_{OC}^{2}-R_{OS}^{2}\right)}}$$

where $R_{OS}$ is outer radius of the steel ring and $R_{IS}$ is inner radius of the steel ring; $R_{OC}$ is the exterior circumference of the concrete ring; and $\nu_{c}$ and $\nu_{s}$ are the Poisson’s ratios of concrete and steel, respectively.

### 5.4. Analytical Models That Predict the Risk of Cracking at Early Ages

The risk coefficient, determined by the RRT, is employed to evaluate the potential for early-age concrete cracking. It can be expressed as the ratio of time-dependent tensile stress ($\sigma_{act}$) to the time-dependent tensile strength ($f_{t}$), as shown in Equation (3).

(3) $$R\left(t\right)=\frac{\sigma_{act}\left(t\right)}{f_{t}\left(t\right)}$$

A value of 0 for the risk coefficient indicates no evolution of tensile stress in the mixture, preventing the occurrence of cracking. As the risk coefficient increases with the gradual development tensile stress, the risk of cracking also rises. In the work by Khan et al. [53], for GGBFS concretes with strengths greater than 40 MPa, the cracking time was slightly greater than that of the reference concretes. Moreover, the 60% GGBFS concrete mix cracked earlier than the 40% GGBFS mixture because the tensile strength of the concrete with 60% GGBFS was less than that of the concrete with 40% GGBFS. Among the tested mixes, the FA concretes exhibited the best performance. This is because the tensile creep and tensile strength of FA concrete were similar to those of reference concretes, but the lower free shrinkage development of FA concretes led to less tensile stress being generated, and the cracking time was subsequently delayed. Thus, the risk coefficient is a frequently employed parameter to evaluate the potential of concrete cracking at early ages.

## 6. Conclusions and Recommendations

This paper conducts a thorough review of a diverse range of literature associated with cracking in OPC-SCM cement-based concrete at early ages. The experimental investigations into early-age cracking of normal concreate have predominantly centered on time-dependent behaviors, such as crack patterns, creep, and shrinkage. However, there has been a limited comprehensive examination of the combined effects of these factors. Additionally, the environmental advantages of SCMs have garnered research attention, but their specific impacts on concrete cracking at early ages remain inadequately explored. Furthermore, there is a noticeable gap in the study of modeling concrete cracking at early ages, particularly for the prediction of time-dependent behaviors of concrete. Consequently, future research exploration of factors influencing the cracking of FA and GGBFS concrete at early ages can be conducted, with a focus on temperature, tensile creep, shrinkage, tensile stress, and temperature evolution of concrete, as follows:

1. In terms of shrinkage, past research has shown that OPC-SCM concrete mixes have a lower shrinkage rate than ordinary concrete, primarily due to the optimized combination of fine aggregates and high-efficiency water-reducing agents. However, the shrinkage rate of these materials is still relatively high, increasing susceptibility to cracking. Future research should focus on further reducing the shrinkage rate and improving the anti-cracking performance. This can be achieved through optimized selection of raw materials, fine-tuned mix design, and improved construction techniques.

2. In terms of creep, OPC-SCM concrete mixes exhibit significantly better creep performance than ordinary concrete. However, as time passes and as environmental conditions change, creep performance may vary, potentially impacting structural performance. Therefore, future research should focus on the long-term creep performance of OPC-SCM blended cement-based concrete to better assess its impact on structural safety.

3. In terms of thermal cracking, OPC-SCM concrete mixes have a lower cement content and higher early strength, resulting in good resistance to thermal cracking. However, at high temperatures, this material has a larger coefficient of thermal expansion, which can lead to cracking. Future research should explore methods to reduce the coefficient of thermal expansion and enhance the thermal cracking resistance through mix ratio optimization and the addition of external admixtures.

4. Future research should also focus on studying the durability, environmental performance, and compatibility of OPC-SCM concrete mixed with other materials in Portland cement-based concrete. Additionally, establishing and refining relevant design specifications and construction guidelines will facilitate the practical application of this material in engineering projects.

## Figures and Tables

**Table 1:** Required parameters for different shrinkage prediction models.

Parameters	ACI209-R92	fib 2010	GL 2000	B4	AS3600-2018	Eurocode 2
Compressive strength at 28 days		Yes	Yes		Yes	Yes
Elastic modulus of concrete at loading	Yes					
Type of cement	Yes	Yes		Yes		Yes
Curing method	Yes		Yes	Yes		
Age of concrete at loading	Yes	Yes	Yes	Yes	Yes	Yes
Relative humidity	Yes	Yes	Yes	Yes		Yes
Volume to surface ratio	Yes	Yes	Yes	Yes		
Slump	Yes					
Fine aggregate content	Yes					
Air content	Yes					
Shape of section				Yes		
w/c ratio				Yes		
a/c ratio				Yes		
Admixtures				Yes		
Hypothetical thickness					Yes	Yes
Location					Yes	

**Table 2:** Required parameters for different creep prediction models.

Parameters	ACI209-R92	fib 2010	GL 2000	B4	AS3600-2018	Eurocode 2
Compressive strength at 28 days	Yes	Yes	Yes		Yes	Yes
Elastic modulus of concrete at loading	Yes	Yes	Yes	Yes	Yes	Yes
Type of cement	Yes	Yes		Yes		Yes
Curing method	Yes		Yes	Yes		
Age of concrete at loading	Yes	Yes	Yes	Yes	Yes	Yes
Relative humidity	Yes	Yes	Yes	Yes		Yes
Volume to surface ratio	Yes	Yes	Yes	Yes		
Slump	Yes					
Fine aggregate content	Yes					
Air content	Yes					
Shape of section				Yes		
w/c ratio				Yes		
a/c ratio				Yes		
Admixtures				Yes		
Hypothetical thickness					Yes	Yes
Location					Yes	
Nonlinear creep		Yes			Yes	Yes

## Data Availability

Data are contained within the article.
